# Forest management affects seasonal source-sink dynamics in a territorial, group-living bird

**DOI:** 10.1007/s00442-021-04935-6

**Published:** 2021-06-01

**Authors:** Kate Layton-Matthews, Michael Griesser, Christophe F. D. Coste, Arpat Ozgul

**Affiliations:** 1 Department of Evolutionary Biology and Environmental Studies, University of Zurich, Zurich, Switzerland; 2 Department of Anthropology, University of Zurich, Zurich, Switzerland; 3 Department of Biology, University of Konstanz, Konstanz, Germany; 4 Centre for Biodiversity Dynamics, Department of Biology, Norwegian University of Science and Technology, Trondheim, Norway; 5 Norwegian Institute for Nature Research, Tromsø, Norway

**Keywords:** Spatial PVA, Metapopulation, Perturbation analysis, Trait-level analysis, Forest management

## Abstract

The persistence of wildlife populations is under threat as a consequence of human activities, which are degrading natural ecosystems. Commercial forestry is the greatest threat to biodiversity in boreal forests. Forestry practices have degraded most available habitat, threatening the persistence of natural populations. Understanding population responses is, therefore, critical for their conservation. Population viability analyses are effective tools to predict population persistence under forestry management. However, quantifying the mechanisms driving population responses is complex as population dynamics vary temporally and spatially. Metapopulation dynamics are governed by local dynamics and spatial factors, potentially mediating the impacts of forestry e.g., through dispersal. Here, we performed a seasonal, spatially explicit population viability analysis, using long-term data from a group-living territorial bird (Siberian jay, *Perisoreus infaustus*). We quantified the effects of forest management on metapopulation dynamics, via forest type-specific demography and spatially explicit dispersal, and how forestry impacted the stability of metapopulation dynamics. Forestry reduced metapopulation growth and stability, through negative effects on reproduction and survival. Territories in higher quality natural forest contributed more to metapopulation dynamics than managed forests, largely through demographic processes rather than dispersal. Metapopulation dynamics in managed forest were also less resilient to disturbances and consequently, may be more vulnerable to environmental change. Seasonal differences in source-sink dynamics observed in managed forest, but not natural forests, were caused by associated seasonal differences in dispersal. As shown here, capturing seasonal source-sink dynamics allows us to predict population persistence under human disturbance and to provide targeted conservation recommendations.

**Supplementary Information:**

The online version contains supplementary material available at 10.1007/s00442-021-04935-6.

## Introduction

Wildlife populations are facing a plethora of threats as a consequence of human activities, which are altering and degrading natural ecosystems (Tilman et al. 2017). Commercial forestry alters forest composition, reduces habitat quality (i.e., habitat degradation), and increases forest fragmentation (Imbeau et al. 2001), with potentially severe impacts on the persistence of natural populations (Noble and Dirzo 1997). Accordingly, forestry management is considered the greatest threat to biodiversity in boreal forests, the largest terrestrial ecosystem. Understanding how populations respond to the effects of commercial forestry, and the implications for their persistence, is therefore imperative for their conservation (Rahmstorf and Coumou 2011; Thompson et al. 2013).

Pulliam (1988) and others (Howe et al. 1991; Dias 1996) used the ‘source-sink’ concept to quantify the demographic consequences of varying habitat quality. Following this concept, several approaches to modelling source-sink dynamics have been developed (Thomas and Kunin 1999; Figueira and Crowder 2006). Population viability analyses (PVAs) provide important conservation tools to predict population persistence under environmental change (Boyce 1992; Akçakaya 2000; Hanski and Gaggiotti 2004). Spatial PVAs have been used to assess how habitat degradation and fragmentation affects population persistence and extinction risk (Doak 1989; Lamberson et al. 1992; Akçakaya and Atwood 1997). Spatial PVAs therefore represent a potentially effective conservation tool, to identify source sites that support less productive sites, although source versus sink characteristics of a given patch can fluctuate in time (Akçakaya et al. 1995; Ozgul et al. 2009).

Temporal environmental stochasticity can lead to fluctuations in population sizes and thus a populations extinction risk (Lande 1993; Fagan et al. 2001). However, seasonality (i.e., a form temporal variability) is not often explicitly accounted for in PVA frameworks, even though most natural populations live in seasonal environments, leading to cyclical changes in vital rates (Fretwell 1972; Norris and Marra 2007). Seasonality may have important consequences for population dynamics (Kot and Schaffer 1984; Stenseth et al. 2003), especially in spatially structured populations, which are driven by stochastic local dynamics and spatial factors (e.g., dispersal), which can both vary seasonally (Paniw et al. 2019, e.g., Behr et al. 2020).

Prospective and retrospective perturbation analyses are commonly used to quantify the relative importance of vital rates for a population’s fitness (Demetrius 1969; Goodman 1971). Later developments extended the application of perturbation analysis to spatially structured populations (Hunter and Caswell 2005), allowing us to quantify the relative importance of local populations for metapopulation dynamics (Ozgul et al. 2009). Such perturbation analyses are similar to the assessment of ‘patch values’, i.e., the contribution of a local population to metapopulation dynamics (Hanski and Ovaskainen 2000). However, perturbation analyses only focus on the largest eigenvalue (i.e., the asymptotic population growth rate), whereas the ratio between the two largest positive eigenvalues or ‘damping ratio’ (Koons et al. 2005) can provide information on a populations resilience to changes in underlying rates. Although damping ratios (and similar concepts) have been used in the context of metapopulation occupancy models (e.g., Day and Possingham 1995; Bode et al. 2008; Shima et al. 2010), they have not applied to spatially structured population projection matrices.

Here, we developed a metapopulation model, combining periodic (Caswell and Trevisan 1994) and vec-permutation matrix approaches (Hunter and Caswell 2005). We implemented environmental fluctuations in metapopulation dynamics via seasonal vital rates. We applied this approach to a population of Siberian jays, *Perisoreus infaustus,* a social bird species, where groups occupy year-round stable territories in boreal forests (Ekman and Griesser 2016). In boreal forests, birds constitute the majority of terrestrial vertebrate species, making this a representative group to focus conservation efforts (Niemi et al. 1998). Territories can be considered as ‘patches’ within a metapopulation since they tend to maintain a stable location among years. They also exhibit asynchrony in local demography and partial dispersal among territories, following the definition of a metapopulation (Fryxell 2001). The study population inhabits two distinct areas within a heterogeneous landscape; one is located in pristine (‘natural’, i.e., not managed for the previous 200 years) forests while the other is located in managed forest consisting of a matrix of heavily managed patches intermixed with few unmanaged patches. Commercially managed forest is generally less dense than natural forest, as the understory is removed several times over a growing cycle. This increases adult predation risk by visually hunting hawks and owls, and also nest predation risk by other corvids (Griesser et al. 2006, 2007, 2017). Consequently, forestry management increases mortality and reproductive failure in Siberian jays (Griesser et al. 2007; Layton-Matthews et al. 2018). By modelling the dynamics of a Siberian jay metapopulation (70 territories), located in natural and managed forest, we quantified the effects of forestry on seasonal metapopulation dynamics and metapopulation stability.

## Materials and methods

### Study system

Data were collected in an individually colour-ringed population of Siberian jays, in northern Sweden (65°40' N, 19°10' E). The study site, near Arvidsjaur, is located in boreal forests dominated by Scots pine (*Pinus silvesteris*) and Norway spruce (*Picea abies*). The site is separated into two areas (south and north) with differing forest management. Forests in the southern area are managed, which involves repeated thinning, harvesting and replanting in 80–120 year-long cycles, resulting in more open, even-aged forest (Griesser et al. 2007; Griesser and Lagerberg 2012). Forests in the northern area have not been managed in the last 200 years and are structurally more diverse and unevenly aged. Hereon, natural forest territories refer to the northern area consisting of predominantly pristine forest (i.e., not managed for the past 200 years). Territories in managed forest included those that were subject to forest management during the study period, where forest management refers to both thinning and clearcutting practices (Griesser et al. 2007).

We used data collected between 2000 and 2014, from 70 territories in natural (*n* = 28) and managed (*n* = 42) forest (see Appendix S1, Table S1 for annual sample sizes). Siberian jays live in family groups generally including a monogamous breeding pair, and 1 − 5 retained offspring and unrelated non-breeders (average group size = 3.5, range = 1 – 7, based on field observations). Retained offspring remain with their parents for up to four years and force their subordinate siblings to disperse from their natal territory soon after fledging. Dominance is reflected in the hatching order, where first hatched individuals have a competitive advantage (Ekman and Griesser 2016). Parents provide retained offspring with nepotistic care, resulting in higher lifetime fitness for individuals delaying dispersal (Ekman et al. 1999; Ekman and Griesser 2002). Breeding occurs in April–May, and subordinate juveniles (i.e., early dispersing juveniles) leave their natal territory during their first summer. All territories were visited repeatedly in March, before the breeding season, and in September, after juveniles have dispersed and settled in another group. Social information was used to determine the rank of all group members (Griesser et al. 2015). We determined the territory core based on nest locations collected from 2000 to 2004 and 2011 to 2013, since Siberian jays appear to focus their time in the area core to the breeding site and territories locations are generally stable across years (Nystrand et al. 2010).

### Local population dynamics

**Fig. 1:**
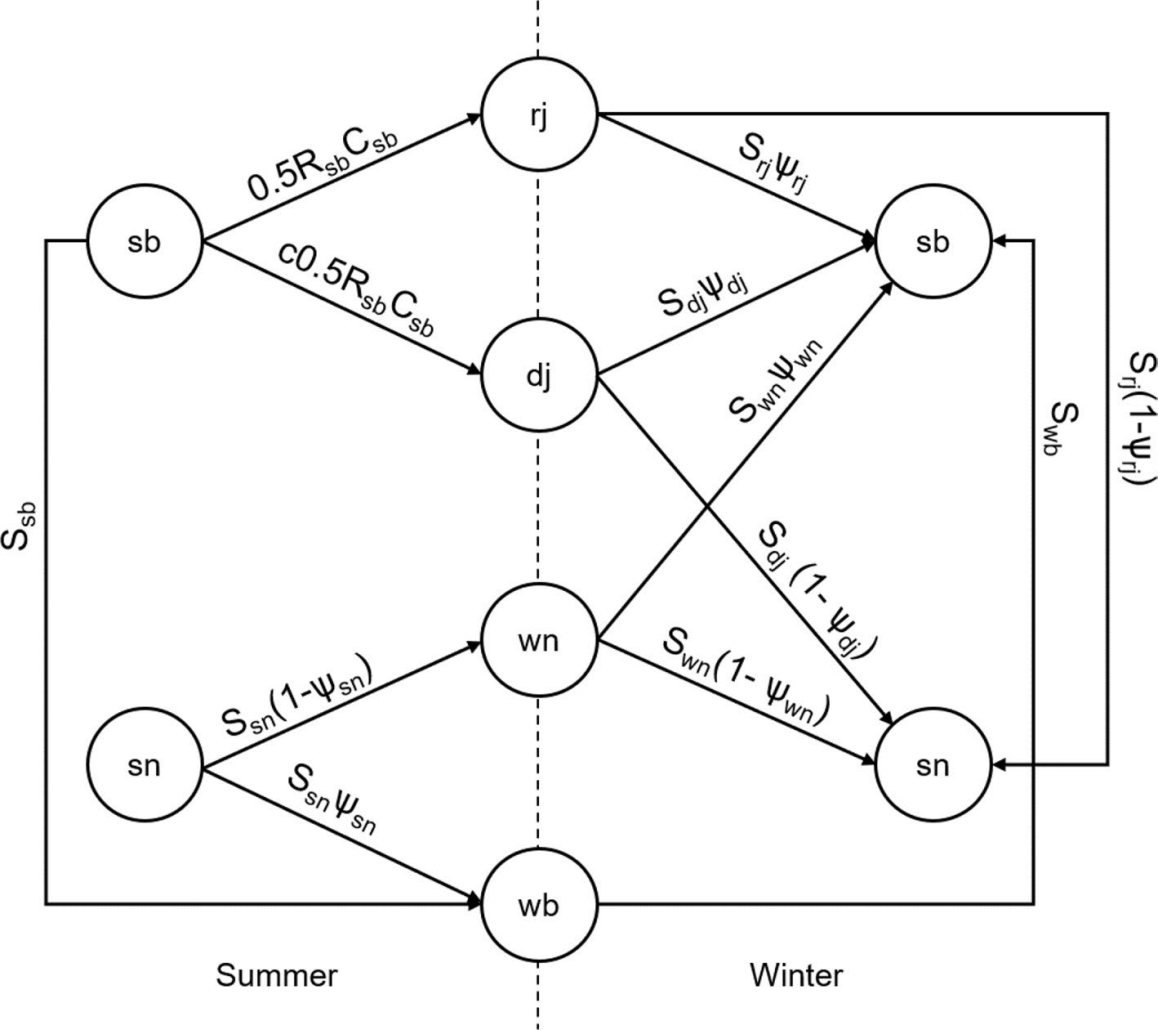
Siberian jay life cycle with life-history stages; retained juvenile (rj), dispersed juvenile (dj), summer non-breeder (sn), winter non-breeder (wn), summer breeder (sb) and winter breeder (wb). The vertical dashed line separates the summer and winter seasons. *S*_*x*_ is the probability of an individual in stage *x* surviving and *Ψ*_*x*_ is the probability of an individual in stage *x* transitioning to a breeder stage at the next census. *R*_sb_ is the probability of a breeding pair producing an offspring and *C*_sb_ is the number of offspring per breeding pair that remains in their natal territory as a retained juvenile. Dispersed juvenile recruitment is retained juvenile recruitment multiplied by *c* (*Methods*)

We used previously estimated seasonal vital rates to describe demography in natural and managed forests. Vital rates were estimated separately for juveniles (new recruits observed in winter), non-breeding and breeding individuals. Life-history stages were also on a seasonal-basis: divided into a summer (March–August) and winter (September–February) phase (Appendix S2, Layton-Matthews et al. 2018). The summer season encompasses the breeding season and main period of juvenile dispersal, while winter is outside the breeding season and dispersal is more limited during this period. Since breeding pairs represent dominant individuals in a social group and provide extended nepotistic care to their offspring, non-breeder/breeder stages apply throughout the year. Transitioning to a breeder only occurs when a breeding position becomes vacant. Consequently, vital rates were specific to the following life-history stages; dispersed juvenile (dj, i.e., individuals that disperse from the natal territory soon after fledging), retained juvenile (rj, i.e., retained offspring that remain in their natal territory), summer (sn) and winter (wn) non-breeder and summer (sb) and winter (wb) breeder (Fig. 1).

Survival (*S*_dj,rj,sn,wn,sb,wb_), recruitment (*R*_sb_, *C*_sb_) and transition rates (i.e., transition probability to a breeding stage conditional on survival, *Ψ*_dj,rj,sn,wn_) were based on the life cycle in Fig. 1. Recruitment described the number of retained juveniles per (summer) breeder in September and was based on two parameters: recruitment rate (*R*_sb_, proportion of summer breeders recruiting a juvenile into the retained juvenile stage) and number recruited (*C*_sb_, number of retained juveniles recruited per summer breeder). Recruitment of retained juveniles was equal to *R*_sb_ × *C*_sb_ × 0.5, to account for female-based reproduction. Recruitment of dispersed juveniles was further multiplied by a correction factor (*c*) to account for 20% dispersal mortality (Griesser et al. 2014) and the proportion of juveniles dispersing (Pd_j_, following paragraph).

Following Layton-Matthews et al. (2018), we parameterised two periodic, stage-structured, population matrices (**K**_summer_, **K**_winter_), which describe demographic processes from summer to winter and from winter to summer, for natural and managed forest (Caswell and Trevisan 1994; Caswell 2001). The winter matrix (right, **K**_winter_) projected the population from the two summer stages (sn and sb) onto four winter stages (rj*, *dj*, *wn and wb). The summer matrix (left, **K**_summer_) projected the four winter stages (rj*, *dj*, *wn and wb) onto the two summer stages (sn and sb).
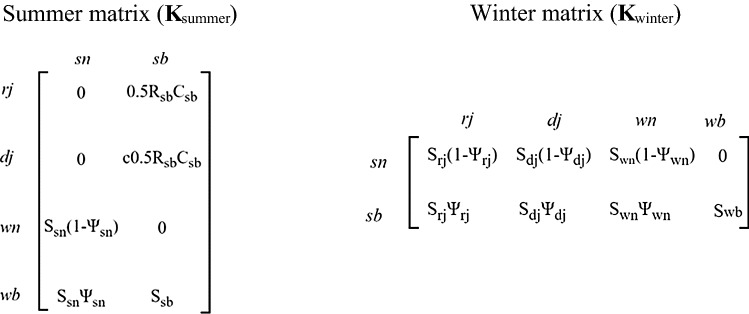


The product of these periodic matrices, **A**, projects the population through an entire annual cycle.

1 $$n\left( {t + 1} \right) = \left[ { {\bf {K}}_{{{\text{summer}}}} \bf {K}_{{{\text{winter}}}} } \right]n(t) = \user2{\bf {A}}n(t)$$

where the population vector, *n*(*t*), gives the density of each life-history stage, in the season at which the projection started.

### Metapopulation modelling

#### Dispersal

Developing a spatial model requires knowledge of dispersal processes. Dispersal events occurred when an individual was recorded in a different territory at season *t* + *1* from season *t*. Data on dispersal events were based on mark-recapture data (seasonal observations of individuals in a given territory) and additional radio-tagging data of dispersed juveniles (to determine the location of juveniles in season *t*). In Siberian jays, dispersal is strongly life history stage-specific and varies between natural and managed forests (Griesser et al. 2014). We estimated dispersal rates (Pd*,* i.e., proportion of individuals dispersing) and tested whether rates were life-history stage and forest-specific, by fitting the data as a binomial distribution and used AICc-based model selection to select the most parsimonious model (Table S1, Appendix S3). We modelled dispersal distance (*d*) as the distance between two territory cores. Data were fitted with five common density functions (i.e., dispersal kernels functions, Clark et al. 1999; Hastings et al. 2005; Van Houtan et al. 2007). We identified the best-fitting kernel and differences between life-history stage and forest type using AICc-based model selection (Table S2, Appendix S3).

#### Spatial population model construction

Equation (1) can also be used to describe a spatial matrix model, where the population vector (*n*) includes the densities of life-history stage *s* in each local population (referred to as ‘patch’, *p*). This spatial (metapopulation) projection matrix, **A**_MP_, incorporates patch and stage-specific demographic and dispersal rates. Spatial matrix models require three components: the state of the metapopulation (state- and patch-specific densities), the demographic processes in each patch, dispersal of individuals among patches (Hunter and Caswell 2005). In this case, **A**_MP_, projects the metapopulation from winter to summer and vice versa, based on local population matrices.

Here, the metapopulation state or population vector, *n*_*i*,*j*_(*t*), is described by the density at stage *i* and patch *j*, where the sub-vectors give the stage distribution within each patch.

We modelled demography without dispersal using the demographic projection matrix **B**, based on the seasonal population matrices (**K**_summer_, **K**_winter_). For each season, **B** is a block diagonal matrix, ordered by patches. The *i*th block of **K**_winter,*i*_ is a 2 × 4 matrix and **K**_summer*,i*_ is a 4 × 2 matrix.

2 $$|n_{1} \vdots n_{p} |_{t + 1} = \underbrace {{\left[ {\begin{array}{*{20}c} {{\bf{K}}_{1} } & \cdots & 0 \\ {} & \ddots & {} \\ 0 & \cdots & {{\bf{K}}_{p} } \\ \end{array} } \right]}}_{{\bf{B}}}|n_{1} \vdots n_{p} |_{t}$$

We modelled dispersal (based on estimated dispersal rates, Pd) using the dispersal projection matrix **M**, for each season (winter and summer). **M** is a block diagonal matrix, ordered by (winter or summer) stages, where the *i*th block **L**_*i*_ is a *p* × *p* matrix describing dispersal rates between patches,

3 $$\, |n_{1} \vdots n_{s} |_{t + 1} { = }\underbrace {{\left[ {\begin{array}{*{20}c} {{\bf{L}}_{1} } & \cdots & 0 \\ {} & \ddots & {} \\ 0 & \cdots & {{\bf{L}}_{s} } \\ \end{array} } \right]}}_{{\bf{M}}}|n_{1} \vdots n_{s} |_{t}$$

where *s* is the number of stages. The sub-diagonal of **M**_winter_ was used to re-distribute retained juveniles to the dispersed juvenile stage, based on the juvenile dispersal rate (Pd_j_)*.*

With this approach, we assumed that demography and dispersal occurred sequentially, with demography occurring first. To conserve the block diagonal forms of **B** (ordered by patches) and **M** (ordered by stages)**,** we used a vec-permutation matrix (**P**_*s*,*p*_) to convert a population vector organised by patches $\left( {\left| {n_{1} \vdots n_{p} } \right|} \right)$ to one organised by stages $\left( {\left| {n_{1} \vdots n_{s} } \right|} \right)$ and vice versa (see Hunter and Caswell 2005 for details). This metapopulation projection matrix (**A**_MP_) can be written as

4 $$\left| {n_{1} \vdots n_{p} } \right|_{t + 1} = \underbrace {{{\bf{P}}_{{{\text{winter}}}}^{T} {\bf{M}}_{{{\text{summer}}}} {\bf{P}}_{{{\text{winter}}}} {\bf{B}}_{{{\text{summer}}}} {\bf{P}}_{{{\text{summer}}}}^{T} {\bf{M}}_{{{\text{winter}}}} {\bf{P}}_{{{\text{summer}}}} {\bf{B}}_{{{\text{winter}}}} }}_{{{\bf{A}}_{{{\text{MP}}}} }}\left| {n_{1} \vdots n_{p} } \right|_{t} ,$$

where $|{n}_{1}\vdots {n}_{p}{|}_{t}$ is the initial metapopulation vector, ordered by patch at season *t*, and ${n}_{p}$ is the population vector for the *p*th patch ordered by stage. **P**_summer_ and **P**_winter_ convert the population vector from a one ordered by patches to one ordered by stages during summer and winter respectively and vice versa for the corresponding transposes (**P**_season_^*T*^).

#### Forest model

We first constructed a spatial population model based on two local populations (i.e. *p* = 2), one occupying natural forest and the other in managed forest (hereon referred to as the ‘Forest model’). **B**_winter_ is a 4 × 8 matrix describing transitions from two winter stages to four summer stages, for each patch (**K**_*p* = 2_). **B**_summer_ is an 8 × 4 matrix (four winter stages to two summer stages). **M**_winter_ is an 8 × 8 matrix where the *i*th diagonal block **L**_*i*_ is a 2 × 2 matrix for each of the four winter stages. **M**_summer_ is a 4 × 4 matrix for each of the two summer stages.

#### Territory model

Second, we constructed a spatial population model at the territory level (i.e. 70 territories/patches) with spatially explicit dispersal, hereon referred to as the ‘Territory model’. Consequently, for the demographic matrix **B**, **K**_*p* = 70_ and the dimensions of **L**_*i*_ are 70 × 70 for stage *i*. To implement spatially explicit dispersal, individuals were forced to disperse within **A**_MP_ at each time step (season), based on distance-dependent dispersal assuming a closed system. Specifically, we quantified the proportion of individuals dispersing into each distance class, based on distance- and stage-specific dispersal distances. We used a lognormal model (Hanski and Thomas 1994; Tischendorf and Fahrig 2000) with *D*_*ij*_ as the response variable describing the number of individuals in patch *i* moving to patch *j.*

5 $$D_{ij} = \frac{1}{{d_{ij} \sqrt {2\pi \sigma^{2} } }}e^{{ - \frac{{\ln \left( {d_{ij} - \mu } \right)_{2} }}{{2\sigma^{2} }}}} ,$$

where *d*_*ij*_ is the midpoint of each distance class and *μ*_*x*_ and *σ*_*x*_ correspond to the mean and variance in dispersal distances, respectively (Moilanen 2004).

### Analyses

#### Metapopulation growth

We calculated asymptotic local population growth rates (natural and manged forest) and the metapopulation growth rate, λ_MP_, (i.e., the asymptotic growth rate across all territories). We estimated confidence intervals for λ_MP_, using a life-table simulation analysis (Wisdom et al. 2000) by simulating 10,000 replicate projection matrices of **A**_MP_, using demographic and dispersal rates sampled from their statistical distributions (Table 1).

**Table 1:** Abbreviations and descriptions of parameters used to describe, seasonal-, forestry- and life history stage-specific demography and dispersal

Rate	Description	Categorisation
*S*_dj,rj,sn,wn,sb,wb_	Apparent survival probability for each seasonal (winter and summer), life-history stage	Demography
Ψ_dj,rj,sn,wn_	Probability of juveniles or non-breeders transitioning to a breeding stage conditional on survival	
*R*_sb_	Probability of a summer breeder recruiting a retained juvenile into a winter juvenile stage	
*C*_sb_	Per capita number of retained juveniles recruited per summer breeder	
*Pd*	Probability of dispersing from a territory	Dispersal
*d*	Distance dispersed	

#### Parameter-level perturbation analysis of the Forest model

We applied a lower-level prospective perturbation analysis to the *Forest model* (Table 2), to determine the relative influence of forest-specific demographic and dispersal rates on λ_MP_. Elasticities were determined analytically using the chain rule for periodic matrices (Caswell and Trevisan 1994; Lesnoff et al. 2003). We performed a fixed, one-way life-table response experiment (LTRE), a common retrospective perturbation analysis (Caswell 1989), to quantify the relative contributions of demographic and dispersal rates to the difference in λ_MP_ between natural and managed forest. In this analysis, the contribution of vital rate *x* to the difference in λ_MP_ was the product of the difference in *x* between natural and managed forest, and the sensitivity of λ_MP_ to *x* (calculated for the natural forest matrix).

**Table 2:** Summary of model structures and analyses applied

Model	Description
*Forest model*	2 forest/patch matrix model describing local dynamics in natural and managed forest, with spatially implicit (stage specific) dispersal **B**_*x*_: Block-diagonal matrices describing demographic transitions in natural and managed forest, for season *x.* Winter matrix projects population from 2 summer stages to 4 winter stages (dimensions = 4 × 8) and vice versa for the summer matrix (8 × 4) **K**_p_: Demography matrix for each patch. Dimensions are 2 × 4 projection matrix for the winter projection and 4 × 2 for the summer projection **M**_*x*_: Block-diagonal matrices describing dispersal between natural and managed patches, for the 4 winter stages (**M**_winter_ = 8 × 8) and 2 summer stages (**M**_summer_ = 4 × 4) **L**_*s*_: 2 × 2 dispersal matrix for each stage for **M**_winter_ (*s* = 4) and **M**_summer_ (*s* = 2) **P**_*x*_ (**P**^T^_*x*_): Vec-permutation matrix and its transpose for season *x.* (**P**_winter_ = 4 × 2, **P**_summer_ = 2 × 2)
*Territory model*	70 territory/patch matrix model, with forest-specific demography and spatially explicit (distance-dependent) dispersal among territories **B**_*x*_: **B**_winter_ = 140 × 280 and **B**_summer_ = 280 × 140 **K**_p_: winter = 2 × 4, summer = 4 × 2 **M**_*x*_: **M**_winter_ = 280 × 280 and **M**_summer_ = 140 × 140 **L**_*s*_: 2 × 2 dispersal matrix for each stage **P**_*x*_ (**P**^T^_*x*_): Vec-permutation matrix and its transpose for season *x* (**P**_winter_ = 4 × 70, **P**_summer_ = 2 × 70)

Analysis	Description
*Parameter-level perturbation*	Prospective (elasticity) and retrospective (LTRE) perturbation analyses of the *Forest model*, to calculate parameter level elasticities and contributions to the difference in λ between natural and managed forests
*Territory-level elasticity*	Using the *Territory model*, elasticities of λ to demography and dispersal (see categorization in Table 2) for each of the 70 territories
*Trait-level*	Using the *Territory model,* the relative importance of dispersal on the stability of the projection matrix, by comparing the damping ratios (Appendix S5) of the managed and natural components of the matrix

#### Territory-level elasticity analysis of the Territory model

Like the *Forest model*, we calculated elasticities of λ_MP_ to lower-level parameters in each patch (i.e., territory). However, here we summed the elasticities to demography and dispersal parameters (see allocation in Table 1), to give the relative influence of demographic and dispersal in each patch on metapopulation growth, for the summer and winter projections (Appendix S4).

We also calculated the connectivity of each patch, to determine the relationship between a patch’s connectivity to neighbouring patches and its influence on metapopulation growth (λ_MP_). Following Moilanen (2004), a patch’s connectivity, *C*_*i*_, was calculated as $C_{i} = \sum\nolimits_{j \ne i} {O_{j} \left( t \right)D\left( {d_{ij} ,\alpha } \right)A_{i} }$. Connectivity of focal patch *i* was the sum over all neighbouring patches *j* of the product of the occupancy statuses, *O*_*j*_(*t*) of patch *j* (where 0 = absent and 1 = present), and the effective distance (*D*_*ij*_) between the focal and neighbouring patch, weighted by the number of individuals per patch (*A*_*i*_, i.e., proxy for patch size). The function (*D*_*ij*_) defines the distribution of dispersal distances, where *d*_*i*,*j*_ is the distance between patches *i* and *j* and *α* defines the distributions of dispersal distances, where 1/*α* is the average dispersal distance (Moilanen 2004).

#### Damping ratios

To study the importance of dispersal processes for metapopulation stability, we performed a ‘trait-level analysis’ (Coste et al. 2017) on the metapopulation projection matrix (**A**_MP_) of the *Territory model*. Trait-level analysis can be used to determine the importance of a given life cycle component (e.g., breeding status, stage or spatial location) and, thus, of the underlying process (e.g., dispersal for spatial location). This method measures the relative importance of a category in a projection matrix (here, spatial location) by ‘folding’ the matrix over that category (see Coste et al. 2017). Using this approach, we calculated the damping ratio (*ρ*), i.e., the ratio of the dominant eigenvalue (λ_1_) to the second largest (sub-dominant) eigenvalue (λ_2_) (Caswell 2001). In this context, the damping ratio is a measure of the resilience of metapopulation growth to changes in dispersal and/or demography, where the *lower* the damping ratio, the more resilient the population is (see Appendix S5 for details on the methodological approach and a simplified example). We compared the *ρ* of the full metapopulation matrix, structured by (spatial) location and stage (**A**_MP_), with the same matrix ‘folded’ over location (i.e., structured by stage only, ${\bf {A}}_{\mathrm{MP}}^{\mathrm{fold}}$), to determine the influence of dispersal on metapopulation stability. We also performed the same analysis for natural and managed forests separately (i.e., to test whether the importance of spatial structure differed between forests).

#### Management scenario

Using the territory model, we calculated λ_MP_ for a scenario where 25% of natural forest territories were removed from the metapopulation matrix, i.e., an expansion in forestry management.

## Results

### Local population dynamics

Previously estimated vital rates (from Layton-Matthews et al. 2018) reflected lower reproductive and survival rates in managed forest. However, stage-transition rates for juveniles and non-breeders to the breeder stage were higher in managed forest than in natural forest (Appendix S2). In addition, we modelled dispersal probabilities and dispersal distances. The best model of dispersal probabilities (Pd) included additive effects of forest type and life-history stage (Appendix S3, Table S1). Juvenile dispersal rate (Pd_j_) was significantly higher than non-breeders and breeders. Dispersal rates were higher in managed forest than natural forest (Appendix S3, Fig. S1). Dispersal distances (*d*) were best approximated with a lognormal dispersal kernel and differed between life-history stages since juveniles dispersed further than post-juvenile life-history stages (Appendix S3, Table S2).

Local population growth rates were 1.00 (CIs: 0.97, 1.03) in natural forest and 0.96 (0.93, 0.98) in managed forest, although CIs overlapped, reflecting lower survival and reproductive rates in managed forest (Table 3). Lower survival and recruitment rates in managed forest (and thus reduced *λ*) were, in part, counteracted by higher transition probabilities of juveniles and non-breeders to breeder status (Appendix S2).

**Table 3:** Estimates of population growth rates (λ) and damping ratios (ratio of the two highest eigenvalues)

Term	Description	Estimate (credible intervals)
(Meta)population growth rates		
*λ*_N_	Asymptotic population growth rate in natural forest (across 28 territories in natural forest)	1.00 (0.97, 1.03)
*λ*_M_	Asymptotic population growth rate in managed forest (across 42 territories in managed forest)	0.96 (0.93, 0.98)
*λ*_MP_	Metapopulation asymptotic growth rate (average across 70 territories)	0.98 (0.96, 1.00)
Trait-level analysis		
* ρ* (**A**_MP_)	Damping ratio for metapopulation projection matrix	1.044
$\rho ({\bf {A}}_{\mathrm{MP}}^{\mathrm{fold}})$	Damping ratio for metapopulation projection matrix *structured by stage only* (folded over location)	1.095
$\rho ({\bf {A}}_{\mathrm{N}})$	Damping ratio for projection matrix of natural forest	1.042
$\rho ({\bf {A}}_{\mathrm{N}}^{\mathrm{fold}})$	Damping ratio for projection matrix of natural forest *structured by stage only* (folded over location)	1.118
$\rho ({\bf {A}}_{\mathrm{M}})$	Damping ratio for projection matrix of managed forest	1.062
$\rho ({\bf {A}}_{\mathrm{M}}^{\mathrm{fold}})$	Damping ratio for projection matrix of managed forest *structured by stage only* (folded over location)	1.141

### Metapopulation dynamics

The asymptotic metapopulation growth rate (λ_MP_, i.e., average *λ* across all territories) for the *Territory model* (i.e., with 70 territories and distance-dependent dispersal) was 0.98 (0.96, 1.00), indicating a stationary metapopulation (neither increasing nor decreasing in size) (Table 3). However, λ_MP_ was reduced to 0.97 (0.95, 0.99) when 25% of natural forest territories (7 territories) were removed from the metapopulation.

**Fig. 2:**
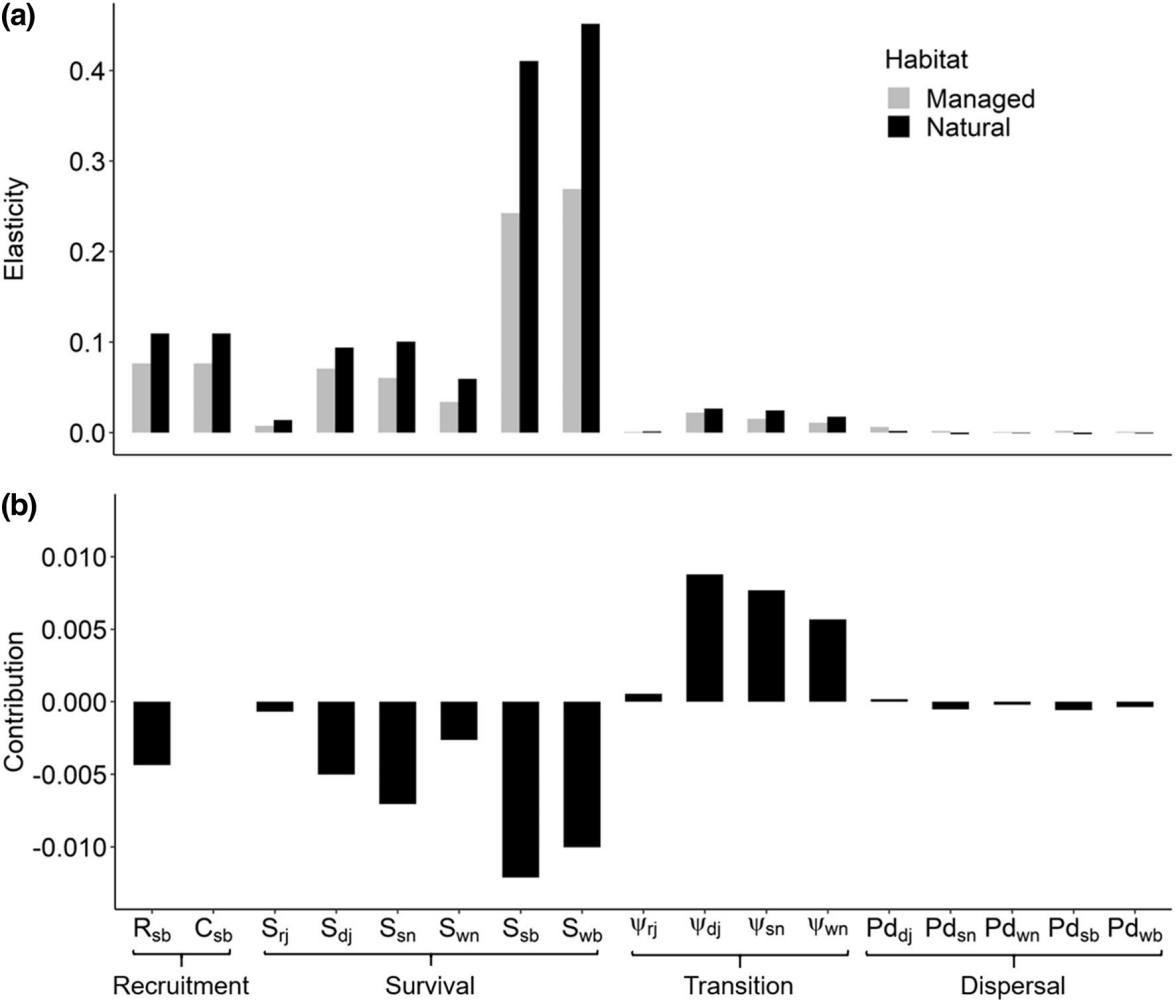
Parameter-level perturbation analysis of the *Forest model:* **a** Elasticities of λ_MP_ to demographic rates; recruitment rate (*R*_sb_), number of recruits (*C*_sb_), life history stage-specific survival (*S*_dj,rj,sn,wn,sb,wb_) and transition probability to breeder (*Ψ*_rj,dj,sn,wn_) dispersal rates (Pd_dj,sn,wn,sb,wb_). Elasticities were calculated using the forest model (i.e., with spatially implicit dispersal), where each patch corresponds to a forest type: managed (grey) and natural (black). **b** Contributions of vital rates to the observed differences in λ_MP_ between natural and managed forest

**Fig. 3:**
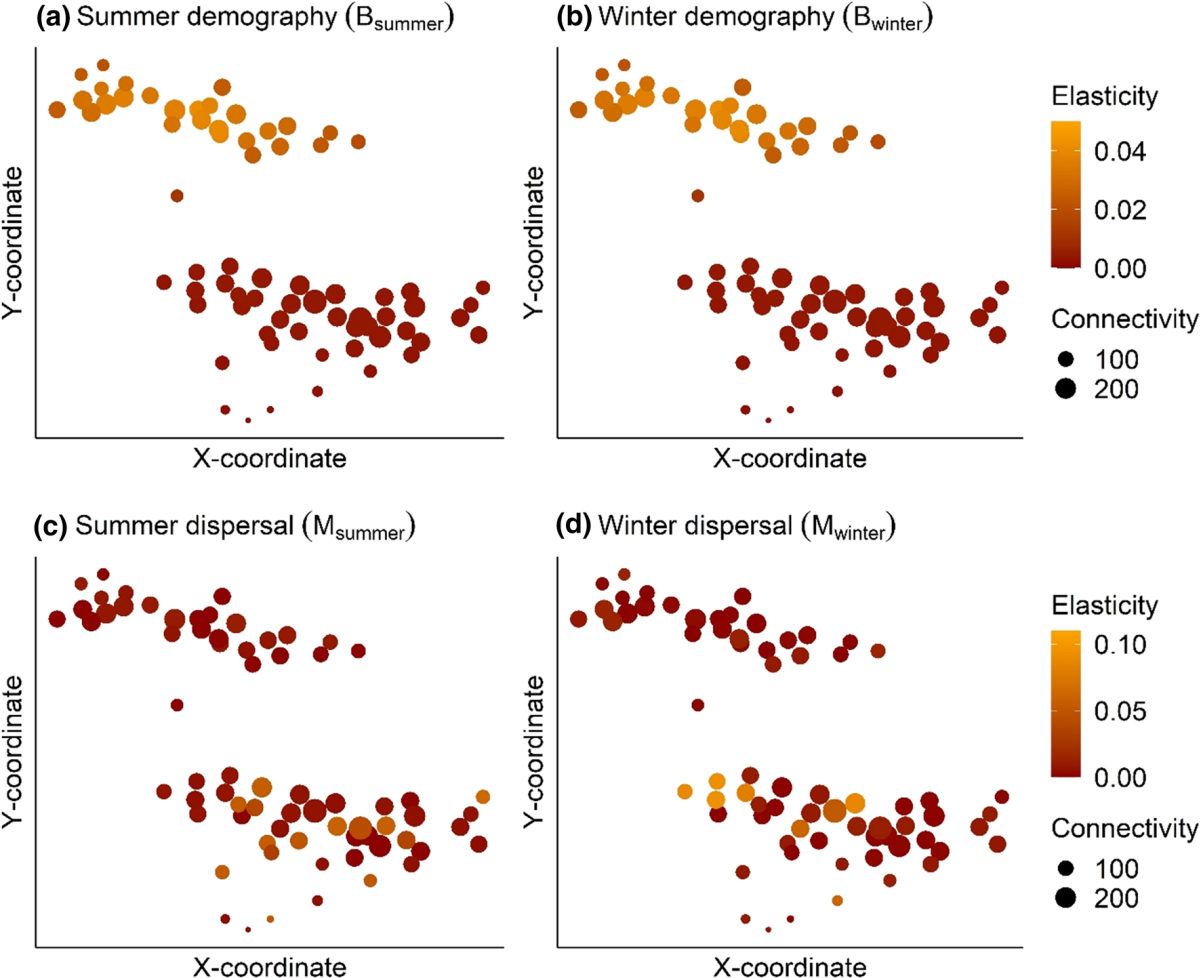
Patch-level elasticity analysis of the *Territory model*: points represent the spatial location of a patch in natural (northern) and managed forest (southern), for a given latitude (*x*-coordinate) and longitude (*y*-coordinate). Patch colour corresponds to the elasticity of λ_MP_ to winter to summer demography (**B**_summer_, **a**), summer to winter demography (**B**_winter_, **b**), summer dispersal (**M**_summer,_ **c**) and winter dispersal (**M**_winter_, **d**), based on the territory model (i.e., spatially explicit dispersal, where one patch = one territory). Patch size corresponds to its connectivity

Metapopulation growth was more sensitive to demography in natural forest than managed forest. (Fig. 2a), but it was more sensitive to dispersal in managed forest than natural forest. Overall, demography had a greater influence on λ_MP_ than dispersal (Fig. 2a). The metapopulation growth rate was most sensitive to breeder survival (*S*_sb_,* S*_wb_), followed by juvenile survival (*S*_dj_, *S*_rj_) and recruitment parameters (*R*_sb_, *C*_sb_) (Fig. 3a). Lower breeding survival and recruitment (*R*_sb_) rates in managed forest were largely responsible for the difference in the contributions of natural and managed forest territories to λ_MP_ (Fig. 3b). However, higher transition rates to breeder status from juvenile and older stages in managed forest increased the metapopulation growth rate in managed forest (Fig. 3b).

### Source-sink dynamics

Based on elasticity analysis of the *Territory model*, the metapopulation growth rate was more sensitive to patch demography in natural than managed forest (Fig. 3). The elasticities of λ_MP_ to demography, summed across all territories in natural forest was 0.031, while it was 0.003 in managed forest territories. Elasticities to demography were similar across seasons and therefore only one estimate is shown. There was a positive relationship between patch connectivity and the elasticity of λ_MP_ to demography in natural forest (Fig. 3a, b). Between forest types, seasonal differences in demography (i.e., reproduction in summer and lower mortality in winter, Appendix S2) had little effect on the influence of patches on metapopulation growth (Fig. 3).

Metapopulation growth was more sensitive to dispersal in managed forest patches (Elas(*M*_*x*_) = 0.020) than in natural forest patches (0.006), and this was consistent across seasons (*x*). Patch connectivity had no influence on the sensitivity of λ_MP_ to dispersal, although there was a large variation in the influence of dispersal on λ_MP_ in managed forest (Fig. 3c, d). There was no seasonal difference in the sensitivity of λ_MP_ to dispersal in natural forest. However, differing rates of dispersal between winter and summer in managed forest (i.e., early dispersing juveniles in winter but fewer non-breeders and breeders dispersing in winter) led to a seasonal change in the distribution of patches with high elasticities. Dispersal had a large influence on λ_MP_ in several managed forest territories, which was not explained by patch connectivity. Further, the identity of these influential territories (i.e., with high elasticities to dispersal rates) differed between seasons (Fig. 3c, d).

### Metapopulation resilience

The damping ratio (*ρ*) for the metapopulation projection matrix (**A**_MP_) was 1.044 $\left( {\frac{{\lambda_{1} }}{{\lambda_{2} }} = \frac{0.984}{{0.943}}} \right)$. *ρ* was higher (1.095) for the folded metapopulation matrix (${\bf {A}}_{\mathrm{MP}}^{\mathrm{fold}}$) because of a lower sub-dominant eigenvalue (*λ*_2_ = 0.899). Consequently, spatial structure reduced the damping ratio by 0.05, indicating that dispersal processes stabilised metapopulation dynamics. The damping ratio for the matrix including only natural forest territories ($\rho ({\bf {A}}_{\mathrm{N}}^{\mathrm{fold}})$ = 1.118) was substantially lower than for the managed forest ($\rho ({\bf {A}}_{\mathrm{M}}^{\mathrm{fold}})$ = 1.141). Consequently, population dynamics in natural forest are more resilient to a disturbance than in managed forest (see Appendix S5). However, the increase in *ρ* between the folded and standard matrices was similar for natural ($\frac{\rho ({\bf {A}}_{\mathrm{N}}^{\mathrm{fold}})}{\rho ({\bf {A}}_{\mathrm{N}})}$ = 1.072) and managed forest ($\frac{\rho ({\bf {A}}_{\mathrm{M}}^{\mathrm{fold}})}{\rho ({\bf {A}}_{\mathrm{M}})}$ = 1.075). Therefore, the stabilising effect of dispersal was similar for both forest types. Thus, the lower damping ratio in natural forest, compared to managed forest, was a result of higher survival and recruitment rates in natural forests.

## Discussion

Successful conservation of species threatened by human activities requires modelling approaches accounting for local dynamics, spatial processes, and temporal variation. Here, we highlight the importance of these processes using a spatial population viability analysis. Forestry management reduced metapopulation persistence and stability in this forest-dwelling bird species. Furthermore, source-sink dynamics varied between forest types, and seasonally, resulting in spatiotemporal variation in the distribution of critical sites for metapopulation dynamics.

### Metapopulation dynamics

Determining extinction risk and metapopulation persistence, and the role of local population dynamics and spatial processes in driving these, is key to the conservation of spatially structured populations (Hanski and Thomas 1994; Keymer et al. 2000; Kahilainen et al. 2018). For this boreal forest-dwelling species, metapopulation growth was stable i.e., overall abundances were neither substantially increasing nor declining over time. Thus, under current levels of forest management, this suggests that the metapopulation should not go extinct, while acknowledging the uncertainties in model parameters. However, when population dynamics were analysed separately for each forest, local population growth rates were stable in natural forest, but declining (*λ* < 1) in managed forest (Table 3). Negative population growth in commercially managed forests was a consequence of lower survival and recruitment. Forests under management have a less heterogeneous structure, leading to increased predation through hawks and owls, which detect prey more easily in more open forests (Griesser et al. 2017).

Under current forest management scenarios, the amount of high quality, pristine habitat will likely decrease further for Siberian jays (Bradter et al. unpublished). Here, we simulated a 25% reduction in natural forest territories, which indicated negative metapopulation growth. This emphasises the importance of retaining remaining pristine forest (e.g., by following more sustainable forestry practices, Spence 2001), to prevent population extinctions in the future.

As is common in longer-lived species, survival of breeding individuals was most influential on population growth and, consequently, was the main contributor to lower population growth rates in managed forest. Breeding individuals are also the dominant members in Siberian jay social groups, with higher survival than non-breeders on average (Griesser et al. 2017). Identifying life-history stages with a large influence on population growth can focus conservation measures. These results indicate that increasing forestry heterogeneity should have a substantial positive impact on population growth, largely via reduced breeder mortality. (Meta)population growth was less sensitive to changes in dispersal rates, compared to survival, transition and recruitment rates (Fig. 2).

### Metapopulation stability

The damping ratio (*ρ*, i.e., ratio of the two largest eigenvalues), and similar concepts, (e.g., Day and Possingham 1995; Bode et al. 2008), has previously been used in a metapopulation context, e.g., as a measure of population-level connectivity (Shima et al. 2010). When applied to metapopulation *projection* matrices, as here, *ρ* provides a quantitative measure of population resilience, where a smaller damping ratio indicates that metapopulation growth is less affected by a disturbance in demographic and/or dispersal rates (see Appendix S5). Using this approach, we measured the role of dispersal in regulating metapopulation dynamics. Despite the comparatively low sensitivity of metapopulation growth to dispersal rates, our analysis of the damping ratio showed that this metapopulation was more resilient to a disturbance, i.e., changes in patch demography or dispersal, because of dispersal processes. Dispersal facilitates the re-allocation of individuals among social groups and reinforces a fixed social hierarchy including early and delayed dispersing juveniles, which consequently exhibit strongly contrasting individual fitness (Ekman et al. 2002; Griesser et al. 2014). In a metapopulation context, although reduced survival or reproduction in high-quality territories reduces metapopulation growth, this is, in part, compensated by poorer-quality territories taking over as ‘engines’ of metapopulation growth, through dispersal. Consequently, dispersal plays a stabilising role by dampening effects of a disturbance in critical, high-quality patches (Strasser et al. 2012). Since territories in natural forest had higher survival and recruitment rates, the resilience of population dynamics in natural forest to a disturbance was greater overall (i.e., a lower damping ratio). This indicates that population dynamics in natural forests are more resilient to a disturbance than in managed forests.

This novel use of damping ratios provides a measure of metapopulation resilience to e.g., local population extinctions, incorporating local dynamics and connectivity using a matrix population framework. This contributes to a better understanding of network properties and their role in mediating effects of (e.g., environmental) changes on metapopulation viability (Gaston et al. 2008). Quantifying metapopulation resilience (Hodgson et al. 2015) has been previously proposed as simple metric for conservation planning, to shift conservation focus from an individual- to multi-site perspective (Donaldson et al. 2019).

### Source-sink dynamics

Source-sink dynamics reflect how differences in habitat quality affect vital rates and, ultimately, local population growth rates (Pulliam 1988). Source-sink dynamics are relatively common in spatially structured populations because of heterogeneity in habitat quality (Dias 1996). Forest-dwelling bird species often live in heterogeneous habitat and evidence of source-sink dynamics exists for such species (Pakkala et al. 2002; Nystrand et al. 2010; Vögeli et al. 2010). Forestry management reduces habitat quality, causing territories in managed forests to more often represent ‘sinks’ (Nystrand et al. 2010).

‘Patch value’ describes the contribution of local populations to metapopulation growth (Ovaskainen and Hanski 2003), which we measured here using territory-level elasticities (Ovaskainen and Hanski 2001, 2003). Overall, patch values were substantially higher in natural forest, compared to territories under forest management, because of the sensitivity of metapopulation growth to changes in demography in natural forests (Fig. 3). This emphasises the preservation of remaining pristine natural forest as a fundamental conservation goal to maintain metapopulation persistence. Furthermore, elasticity analysis showed that the influence of natural forest territories increased with territory connectivity, because they are more able to contribute individuals to surrounding patches (i.e., to act as source). Thus, in natural forest, isolated patches at the edges of the metapopulation contributed least to metapopulation persistence since dispersal is more limited, potentially increasing their extinction risk (Hanski and Ovaskainen 2000; Brito and Fernandez 2002). In managed forest, we identified few critical sites, where metapopulation growth was substantially (up to three times) more sensitive to changes in dispersal, than other managed forest territories. Since these critical sites contribute disproportionately to metapopulation growth, they could represent specific targets for conservation measures (Brito and Fernandez 2002). Preserving connectivity among patches (particularly allowing for dispersal from higher quality natural forest territories), would therefore also present a valuable conservation goal. However, as shown by the damping ratios, lower quality patches (i.e. sinks) can also act to buffer disturbance effects in more productive sites (Howe et al. 1991; Gill et al. 2001; Strasser et al. 2012). Asynchrony in local dynamics, ultimately limits variation in metapopulation growth, which increases metapopulation stability (Hanski 1998). Dispersal is therefore inherently important to facilitate buffering of disturbance effects, including further habitat degradation caused by commercial forestry. Nevertheless, demographic processes in natural forest territories were the main driver of metapopulation growth, while demography in managed forest had a far more minor impact on the growth rate. Conversely, dispersal in several managed forest territories made a large contribution to metapopulation growth, while dispersal in natural forest had a far lesser impact on the growth rate. Furthermore, the influence of dispersal on metapopulation growth in managed forest territories did not correlate with connectivity and there was greater variation among territories in their contributions than in natural forests.

Density dependence (density-dependent demography and dispersal) can be important in driving source-sink dynamics, e.g., where suppressed local recruitment causes a viable local population to be characterise as a sink (Watkinson and Sutherland 1995). The mechanisms by which density influences Siberian jay population dynamics are complex: higher densities positively effect survival and transition rates because of group vigilance. Density also positively effects recruitment rates but as an interactive effect with temperature, reflecting reduced resource competition in warming springs (Layton-Matthews et al. 2018). Due to the complex role of density-dependence, the metapopulation model formulated here was density independent.

Vital rates often vary seasonally, potentially affecting source-sink dynamics and thus metapopulation stability (Boughton 1999; Gill et al. 2001). In our study, seasonal variation in the contribution of demography on metapopulation growth rates was negligible. The sensitivity of λ_MP_ to dispersal in managed forests differed between seasons, likely due to higher dispersal rates in summer, although the contribution of this to metapopulation dynamics was small relative to demographic contributions. Patch values of territories in natural forest differed little between winter and summer, for both demography and dispersal. The minor role of seasonality (i.e., differences in vital rates between seasons) in driving metapopulation dynamics is likely explained by the dynamics of natural forest populations, which are largely driven by breeder survival that exhibited little variation across seasons. Ultimately, seasonality in demography and dispersal rates appear to play a somewhat minor role in the metapopulation dynamics of this particular species.

### Conclusions

Our results show how forest management has reduced metapopulation growth and stability in a boreal forest species. Populations may therefore be increasingly vulnerable to further forestry effects and increasing temporal variability (e.g., due to climate change, Kahilainen et al. 2018). Overall, populations occupying natural forests have higher growth rates and are more inherently resilient to disturbance. Consequently, further loss of territories in natural forest could push such Siberian jay metapopulations towards extinction. Climate change has been shown to increase the difference in population trajectories in natural versus managed forests, thereby exacerbating the risk of metapopulation extinction (Layton-Matthews et al. 2018). These results therefore emphasise the necessity to protect remaining natural, old-growth, forests to conserve this species. Moreover, increasing structural heterogeneity in commercially managed forests should improve survival rates and thereby local population growth rates. Despite the apparent insensitivity of metapopulation growth to dispersal rates (based on perturbation analysis), dispersal plays a key role in metapopulation stability. It is therefore imperative to conserve both high-quality sites and maintain dispersal networks in *both* natural and managed forests. Our study also emphasises the value of studying eigenvalue distributions, particularly in the context of spatially structured populations.

The specificities of Siberian jay life history (e.g., stable territories, a rigid social hierarchy), have likely facilitated the development of such a detailed, spatial PVA. This may limit the generality of our findings to other species, even in boreal forests, as social structure and dispersal behaviour are rather unique to this species. Nevertheless, Siberian jays can, to some extent, be considered as an umbrella species, where the conservation-related recommendations here would likely benefit many other boreal species. Despite, the high-quality data requirements such PVAs have also been performed for other species (e.g., Ozgul et al. 2009). We emphasise the value in utilising increasingly advanced statistical tools to improve population viability assessments and ultimately make better predictions of wildlife population persistence in the face of ongoing human activities.

## Supplementary Information

Below is the link to the electronic supplementary material.

Supplementary file1 (PDF 352 kb)

Supplementary file1 (DOCX 660 kb)

## Data Availability

Data used in this study will be submitted to Dryad.
