# Supplementary material for: Forest management affects seasonal source-sink dynamics in a territorial, group-living bird
Source: Oecologia. 2021 Jun 1;196(2):399–412. doi: 10.1007/s00442-021-04935-6 (PMC8241677; doi:10.1007/s00442-021-04935-6)
Supplement: Supplementary file 1 — Supplementary file1 (PDF 352 kb) [file 442_2021_4935_MOESM1_ESM.pdf]

Supplementary Material: The Damping Ratio as measure of resilience in the context of multi-site demographic models

1 Damping ratio of a stage-structured population

1.1 Transient theory and definitions

We start with some core concepts of transient theory (linear population dynamics) and the definition of the damping ratio in the context of stage-structured population dynamics. Let us consider matrix $\mathbf{M}$, which projects, over time, a population structured by n stages, in a constant environment. Matrix $\mathbf{M}$ is of size n x n, with $M_{i,j}$ the transition rate from stage j to stage i. In most cases of stage structured matrices (Lefkovitch, 1965) the related life cycle is strongly connected (an individual can transition from one stage to another in a finite number of time-steps), rendering the matrix *irreducible* and the Perron-Frobenius theorem thus guarantees that the maximum eigenvalue is real and positive; we denote it λ_1 and it corresponds to the long-term growth rate of the population (Perron, 1907; Frobenius, 1912). Matrix $\mathbf{M}$ has n eigenvalues in total, either real or complex conjugates, that we can sort in decreasing order of their absolute values: $\boldsymbol{s}_{\mathbf{M}} = \{\lambda_1, \lambda_2, ..., \lambda_n\}$. For each eigenvalue, λ_i, there is a corresponding right-eigenvector $\boldsymbol{w}_i$ (scaled to sum to 1) and a left-eigenvector $\boldsymbol{v}_i$ (scaled so $\boldsymbol{v}_i^T \boldsymbol{w}_i = 1$). In particular $\boldsymbol{w}_1$, where $\mathbf{M}\boldsymbol{w}_1 = \lambda_1 \boldsymbol{w}_1$, corresponds to the asymptotic abundance vector and $\boldsymbol{v}_1$, where $\boldsymbol{v}_1^T\mathbf{M} = \lambda_1 \boldsymbol{v}_1^T$ to the vector of reproductive values (as defined by Fisher, 1930). Matrix $\mathbf{M}$ is similar to $\mathbf{D}$, the diagonal matrix, of which the diagonal is the spectrum vector $\boldsymbol{s}_{\mathbf{M}}$, such that $\mathbf{M} = \mathbf{P}^{-1}\mathbf{D}\mathbf{P}$; the rows of $\mathbf{P}$ correspond to the $\boldsymbol{v}_i^T$ and the columns of $\mathbf{P}^{-1}$ to the $\boldsymbol{w}_i$. This implies that we can rewrite the classical population projection equation for population vector at time t, $\boldsymbol{n}_t$, as $\boldsymbol{n}_{t+1} = \mathbf{M}\boldsymbol{n}_t = \mathbf{P}^{-1}\mathbf{D}\mathbf{P}\boldsymbol{n}_t$. Therefore, for an initial population vector $\boldsymbol{n}_0$, we have

$$\boldsymbol{n}_t = \mathbf{M}^t \boldsymbol{n}_0 = \mathbf{P}^{-1}\mathbf{D}^t\mathbf{P}\boldsymbol{n}_0 = \sum_i \lambda_i^t (\boldsymbol{v}_i^T \boldsymbol{n}_0)\boldsymbol{w}_i = \lambda_1^t \sum_i (\frac{\lambda_i}{\lambda_1})^t (\boldsymbol{v}_i^T \boldsymbol{n}_0)\boldsymbol{w}_i. \quad (1)$$

From expression (1), we see the dominant role played by the maximum eigenvalue λ_1 as t increases: $\frac{\boldsymbol{n}_t}{\lambda_1^t}$ then tends towards $(\boldsymbol{v}_1^T \boldsymbol{n}_0)\boldsymbol{w}_1$, as the *components* related to the subdominant eigenvalues $\lambda_2, \lambda_3, etc.$ (by decreasing order of importance) tend towards zero. This consideration suggests a metric for the speed at which a population vector converges towards the stable state abundance vector $\boldsymbol{w}_1$; this is the damping ratio $\rho_M = \frac{\lambda_1}{|\lambda_2|}$ (see Caswell, 2001). Equation (1) shows that one way to interpret the spectrum of population projection matrix $\mathbf{M}$ is as a decomposition of the growth rate and abundance vector into n *components*, the ith of which, has *support* $\boldsymbol{w}_i$ (scaled by factor $\boldsymbol{v}_i^T \boldsymbol{n}_0$) and growth rate λ_i. Asymptotically, $\boldsymbol{w}_1$ and λ_1 take over (since for $i \neq 1$, $\boldsymbol{v}_i^T \boldsymbol{w}_1 = 0$), but in the general case all other components contribute to the dynamics of the population, particularly for those with large $|\lambda_i|$.

Crucially, from expression (1), in the case of $|\lambda_i| > 1$ (rare in stage-structured models for $i \neq 1$, see below), we see that, whilst the *relative* (to component 1) importance of the ith component will tend towards zero as t increases, the *absolute* contribution of that component to the population vector increases with t (as long as $\boldsymbol{v}_i^T \boldsymbol{n}_0 \neq 0$).

1.2 Interpretation and illustration

In the framework of transient dynamics of stage-structured populations, ρ is therefore a measure of the effect of a shift from the relative asymptotic abundances $\boldsymbol{w}_1$ (that can be caused by past environmental perturbations) on short to mid-term dynamics. Let us illustrate this with a simple example. In Figure 1, we plot the annual growth rates of two age-structured models (i.e., as Leslie matrices), which differ in their respective ρ, after a perturbation in the abundance vector. Population A is modelled by Leslie

matrix $\mathbf{A} = \begin{bmatrix} 0.4 & 1.5 & 1.1 \\ 0.8 & 0 & 0 \\ 0 & 0.6 & 0 \end{bmatrix}$ and population C by matrix $\mathbf{C} = \begin{bmatrix} 1.4 & 1.4 & 1.4 \\ 0.16 & 0 & 0 \\ 0 & 0.2 & 0 \end{bmatrix}$. Matrix $\mathbf{C}$ has a higher asymptotic population growth rate $\lambda_{1_C} = \lambda_C = 1.56$ than matrix $\mathbf{A}$ ($\lambda_A = 1.46$). We set $\boldsymbol{n}_0 = [1, 0, 0]^T$, which is different from both $\boldsymbol{w}_{1_A} = \boldsymbol{w}_A = [0.56, 0.31, 0.13]^T$ and $\boldsymbol{w}_C = [0.90, 0.09, 0.01]^T$. Since the subdominant eigenvalues (i.e., $\{\lambda_2, \lambda_3, ..., \lambda_n\}$) of $\mathbf{A}$ (of spectrum $\boldsymbol{s}_{\mathbf{A}} = \{1.46, -0.53{-}0.27i, -0.53{+}0.27i\}$) are closer to the dominant one than those of $\mathbf{C}$ (of spectrum $\boldsymbol{s}_{\mathbf{C}} = \{1.56, -0.08{-}0.15i, -0.08{+}0.15i\}$), the annual population growth rate tends more slowly towards its asymptotic limit (the deterministic growth rate). This is reflected in their respective damping ratios: $\rho_A = \frac{\lambda_{1,A}}{|\lambda_{2,A}|} = 2.44 < \rho_C = \frac{\lambda_{1,C}}{|\lambda_{2,C}|} = 9.22$

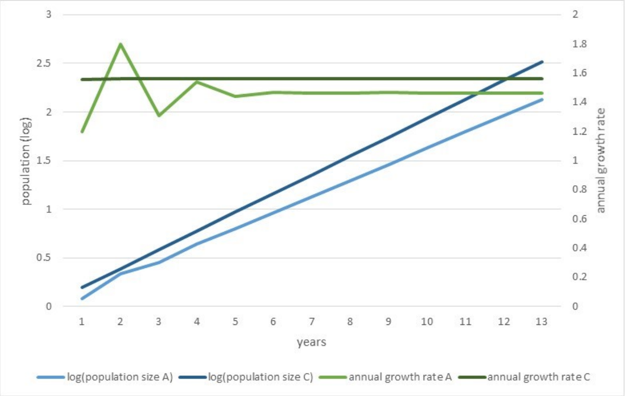

Figure 1: population size and annual growth rate for matrix $\mathbf{A}$ and matrix $\mathbf{C}$ models after perturbation of abundance vector.

Let us note that, in this case, and in most cases of stage-structured models, many eigenvalues are complex (conjugates). This explains the "oscillations" towards the asymptotic growth rate, by contrast to a monotonous convergence obtained with a positive eigenvalue (Holgate and Caswell, 1990). It can actually be shown that, in the case of a Leslie matrix, the only positive eigenvalue is the maximum one (by showing that all assumed positive eigenvalues have the same, because uniquely defined, right-eigen vector of asymptotic abundances; see e.g., Diekmann et al., 2003). These subdominant eigenvalues can therefore hardly be interpreted as "subdominant growth rates", but they still are, when combined (hence the complex *conjugation*), measures of the strength of the projection of population vector towards other *supports* than $\boldsymbol{w}_1$.

2 Damping ratio of a multi-site population

Multi-site models refers to projection matrices that incorporate information on location (e.g., category "patch", "site", "area") as one of their categories, whilst also being structured by "classical" life-cycle categories (e.g., age, stage). For a multi-site model, transitions among states refer to both life history transitions (e.g., survival, fertility, growth) and movement transitions (e.g., dispersal, migration). Multi-site demography therefore combines the frameworks of evolutionary demography (generally forsaking spatial structure) and metapopulation theory (largely based on occupancy models).

2.1 Prospective sensitivity analysis

As mentioned above, ρ measures the effect of a change in the population vector (away from $\boldsymbol{w}_1$) on short/mid-term dynamics, in a *constant* environment. Population ecologists also require instruments to measure the consequences of variation in the environmental conditions on *long-term* dynamics. This is provided by another set of tools, commonly referred to as perturbation analysis. In order to understand the effect of an environmental change (through its effects on vital rates) on the long term growth rate (λ_1) one can, for instance, consider the *sensitivity matrix*, $\mathbf{S} = \boldsymbol{v}_1 \boldsymbol{w}_1^T$, where $S_{i,j}$ corresponds to the sensitivity of λ_1 to changes in $M_{i,j}$: $S_{i,j} = \frac{\partial \lambda_1}{\partial M_{i,j}}$, i.e. to the *infinitesimal* change in λ_1 caused by an *infinitesimal* change in $M_{i,j}$.

For models whose spectrum is not *compact* (subdominant eigenvalues are distant from λ_1) as for stage-structured populations, this tool can be extended to interpret the consequences of more biologically meaningful changes in vital rates (i.e. not infinitesimal). Indeed, in such models, non-infinitesimal changes in vital rates will still preserve λ_1 as the maximum eigenvalue even if it is reduced. It may

prove insufficient, however, for more complex projection models, where the spectrum is more *compact* and where subdominant eigenvalues can have direct ecological interpretations as "subdominant growth rates", as in the case of multi-site projection matrices. In such cases, the dominant position of λ_1 is not guaranteed since (even small) changes in vital rates, can lead to λ_2, or other subdominant eigenvalues, becoming dominant.

2.2 Properties of multi-site models

Multi-site matrix models, are rarely *irreducible* (some *state* x *location* combinations cannot be reached from other *state* x *location* combinations). However, we know from theory that any non-negative matrix is similar to its *Frobenius normal form*, which is a matrix of block-triangular form with irreducible sub-matrices as diagonal blocks (Gantmacher, 1959; Varga, 1962). Let us now consider a multi-site *age* x *patch* model already in its *Frobenius normal form*, with block matrices corresponding to transitions within patches.

Let $\mathbf{M} = \begin{bmatrix} \mathbf{A} & \mathbf{D_{B->A}} & \mathbf{D_{C->A}} \\ 0 & \mathbf{B} & \mathbf{D_{C->B}} \\ 0 & 0 & \mathbf{C} \end{bmatrix}$, where $\mathbf{A}$ and $\mathbf{C}$ are as described above and $\mathbf{B} = \begin{bmatrix} 0.5 & 0 & 1 \\ 0.18 & 0 & 0 \\ 0 & 0.4 & 0 \end{bmatrix}$ is the Leslie matrix for patch B, with $\boldsymbol{s}_\mathbf{B} = \{0.66, -0.08 - 0.32i, -0.08 + 0.32i\}$, and where dispersal matrices $\mathbf{D_{K->L}}$ are zero-matrices except for age-1 dispersal rates $D_{2,1 B->A} = 0.42$, $D_{2,1 C->A} = 0.08$, and $D_{2,1 C->B} = 0.16$. In this model, patch C seems to be of better quality (higher internal growth rate) than patch A, followed by patch B (which has a maximum eigenvalue lower than 1). In reality, one should also account for individuals produced by the patches that disperse in order to assess the quality of a patch. We still have $\lambda_{\mathbf{C}+\mathbf{D_{C->B}}+\mathbf{D_{C->A}}} = 1.75 > \lambda_\mathbf{A} = 1.46 > \lambda_{\mathbf{B}+\mathbf{D_{B->A}}} = 0.84$. Using metapopulation vocabulary, patch C is therefore a *source* and patch B a *sink*. Patch A cannot be characterised as a source or sink since it is both growing in size and producing a net positive number of individuals per year, but with a net negative dispersal balance.

By the properties of triangular block matrices, the spectrum of matrix $\mathbf{M}$ is the concatenation (i.e., the 'combination') of the spectra of $\mathbf{A}$, $\mathbf{B}$ and $\mathbf{C}$, that is, $\boldsymbol{s}_\mathbf{M} = \{1.56, 1.46, 0.66, -0.53 - 0.27i, -0.53 + 0.27i, -0.08 - 0.32i, -0.08 + 0.32i, -0.08 - 0.15i, -0.08 + 0.15i\}$. The spectrum still contains complex conjugate eigenvalues but now also comprises of two subdominant, positive (thus real) eigenvalues. The first characteristic stems from its stage structure and the second from its metapopulation structure. In fact, because the corresponding graphs of projection matrices structured by location are more "symmetrical" (i.e., individuals can transition in both directions between patches, which is not possible between e.g., consecutive age classes), spatially-structured matrices have more real eigenvalues than stage-structured models (actually, a symmetrical matrix has all of its eigenvalues real, see Hawkins, 1975). This can be seen by considering the *folded* versions of $\mathbf{M}$ with respect to the *age* category and *patch* category, i.e., matrices that are asymptotically equivalent to $\mathbf{M}$ (here the asymptotic growth is λ_C), but where the population is structured according to only one category (Coste et al., 2017).

Here $\mathbf{M}^{fold}_{age} = \begin{bmatrix} 1 & 1.2 & 1.1 \\ 0.55 & 0 & 0 \\ 0 & 0.49 & 0 \end{bmatrix}$ is the reference Leslie matrix of the population and has spectrum $\{1.56, -0.28 - 0.34i, -0.28 + 0.34i\}$ whilst $\mathbf{M}^{fold}_{patch} = \begin{bmatrix} 1.48 & 0.07 & 0.07 \\ 0 & 0.54 & 0.14 \\ 0 & 0 & 1.56 \end{bmatrix}$ is the matrix of annual dispersal rates, where stage structure is ignored, and its spectrum only contains positive eigenvalues $\{1.56, 1.48, 0.56\}$ on the diagonal.

2.3 Consequences of spatial structure on effects of environmental changes

Here, these positive eigenvalues, contrary to their complex or negative counterparts, correspond to ecological concepts: they are the internal growth rates of the various patches, when accounting for dispersal (and their associated right eigenvectors are also real, albeit not always positive). The patch with the highest growth rate, here patch C, provides the multi-site matrix with its asymptotic growth rate. However, the subdominant real eigenvalues (generally, as we can see here, higher in absolute values that the complex ones) also play a role with regards to the fate of the population in case of long-term changes in the environment.

In the simplified case where a change in vital rates $\delta\mathbf{M}$ only affects the eigenvalues of the dominant

patch, then the change in "population fitness" will only be accurately computed via sensitivity analysis if the new value of maximum eigenvalue of the, so far, dominant patch is still higher that those of other patches. Otherwise, that role will be taken over by another patch and the new "population fitness" will be higher than the new value of the maximum eigenvalue of the, so far, dominant patch. In other words, the more *compact* the spectrum of a multi-site matrix model, the more resilient it is to environmental changes. Such a resilience can be approximated by the ratio of the two maximum eigenvalues, that is, by the ρ in transient analysis.

2.4 Illustration

Following the multi-site example presented above, imagine an alternative environment where patch B and C remain the same but patch A individuals do not reproduce after the first year. This new model, denoted as $\mathbf{M'}$, has the same maximum eigenvalue as $\mathbf{M}$ (i.e., the one of patch C) but because of the reduced fertility in A, the three highest eigenvalues (i.e., the three positive, real eigenvalues corresponding to the "internal growth" rates of each patch) are now equal to $\{1.56, 0.66, 0.4\}$ instead of $\{1.56, 1.46, 0.66\}$. With its subdominant eigenvalue much closer to its current asymptotic growth rate model $\mathbf{M}$ is more resilient to changes (in the current engine of its population growth, patch C, specifically) than is model $\mathbf{M'}$. This can be summarised by computing the ρs: $\rho_{M'} = \frac{1.56}{0.66} = 2.36 > \rho_M = \frac{1.56}{1.46} = 1.07$. For model $\mathbf{M}$, if a change in vital rates led to a ρop in λ_C that was below the current value of λ_A, then λ_A would take over as the asymptotic population growth rate in the new environment, in which the population would still be growing and at an asymptotic rate that is relatively close to the one in the previous environment. In model $\mathbf{M'}$, λ_C has to drop much further for this situation to occur, and by then the metapopulation is already on its way towards extinction.

The difference between these two models would not be captured by perturbation analysis tools, such as elasticity or sensitivity analysis, since these only focus on the current maximum eigenvalue and related eigenvectors (which are the same in both models). However, this is, in part, captured by the lower ρ for $\mathbf{M}$ than for $\mathbf{M'}$, used as a proxy for the compactness of the spectrum of $\mathbf{M}$. We illustrate this in Figure 2, where the dynamics of population models $\mathbf{M}$ and $\mathbf{M'}$ are depicted starting with an initial population consisting of one 1 year-old individual in patch C, and with, in year 14, a change in environment: patch C fertility rate is reduced to 0.4.

Although the model with the lowest ρ, $\mathbf{M}$, converges more slowly towards λ_C (prior to year 14), $\mathbf{M}$ is also more resilient to the environmental change at year 14, with populations growing asymptotically after a transient period where the annual growth rate drops to 1 (rather than for $\mathbf{M'}$, where the growth rate drops to 0.85).

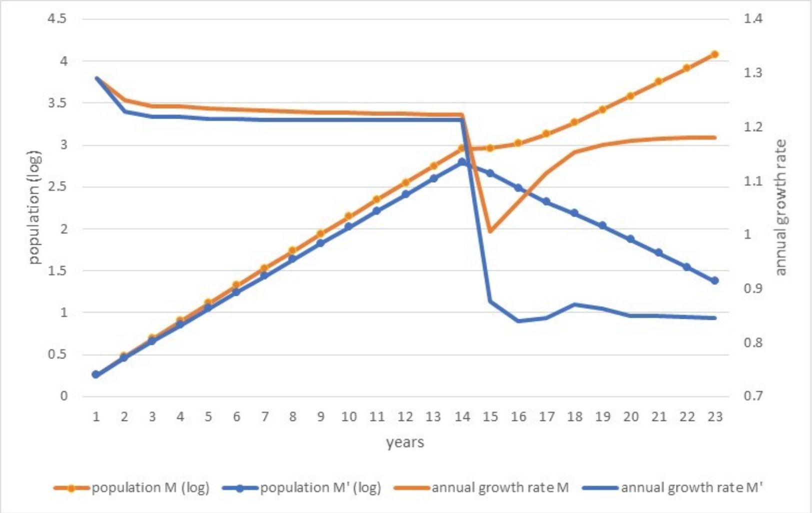

Figure 2: Annual growth rate and total population sizes for models $\mathbf{M}$. and $\mathbf{M'}$, where environmental change (reduction in patch C fertility rate to 0.4) occurs year 14. Initial population is one 1 year-old individual in patch C.

2.5 The damping ratio as a measure of resilience to environmental changes

It may seem rather counter-intuitive that the ratio of the two highest eigenvalues (i.e., the damping ratio) is used here, in the framework of sensitivity analysis of multi-site models, as a measure of resilience of the long-term growth rate to non-infinitesimal changes in the life cycle. Especially so, since we claim that a low ρ implies higher resilience to environmental shifts in the life cycle whilst, in transient theory, it also implies a slower convergence to stable state of the abundance vector after a perturbation, that weakens the population.

nIn fact, the "benefit" in having a large damping ratio seem rather specific to stage-structured models, where the second eigenvalue (often a complex conjugate) generally with an absolute value of less than one, is far from the asymptotic growth rate (spectrum not *compact*). In that case, having a larger damping ratio means spending less time with the population vector being projected by other *supports* than $\boldsymbol{w_1}$ (e.g., $\boldsymbol{w_2}$ or $\boldsymbol{w_3}$) that actually decrease the population size and may therefore be deemed "good" for the population.

However in a general context, and particularly regarding multi-site models, if the compactness of the spectrum is high (implying a low damping ratio), other *supports* than $\boldsymbol{w_1}$ with positive growth rates can take over (temporarily in the context of transient analysis, permanently in the context of prospective sensitivity analysis) when an environmental perturbation occur. As Figure 2 demonstrates, after perturbation of the abundance vector (in year 1), the model with lower damping ratio converges more slowly to the (initial) asymptotic growth rate. However, it does so with higher abundances than the model with a higher damping ratio. Most importantly, it maintains positive growth after a perturbation of vital rates (in year 14) which affects the initial growth rate.

To some extent, ρ measures the compactness of the spectrum of a projection model, that is, how strong other *supports* than $\{\lambda_1, \boldsymbol{w_1}\}$ are. Strong subdominant *supports* (e.g., a high λ_2, implying a low ρ) implies that they will play a role after a perturbation, both by lengthening the time to convergence in the context of transient dynamics (but that time is spent on *supports* that increase the population size, so the effect on the population is actually positive) and by ensuring a stronger resilience to (non-infinitesimal) changes in vital rates.

3 References

Caswell, H. (2001). *Matrix Population Models.* Sinauer Associates Inc., Sunderland, MA.

Coste, C. F. D., Austerlitz, F., and Pavard, S. (2017). Trait level analysis of multitrait population projection matrices. *Theoretical Population Biology*, 116:47–58.

Diekmann, O., Gyllenberg, M., and Metz, J. (2003). Steady-state analysis of structured population models. *Theoretical Population Biology*, 63(4):309–338.

Fisher, R. A. (1930). *The Genetical Theory of Natural Selection.* Oxford University Press.

Frobenius, G. (1912). *Über Matrizen aus nicht negativen Elementen.* S.-B. Preuss Akad. Wiss. Berlin.

Gantmacher, F. (1959). *The Theory of Matrices, Volume 2.* American Mathematical Soc.

Hawkins, T. (1975). Cauchy and the spectral theory of matrices. *Historia Mathematica*, 2(1):1–29.

Holgate, P. and Caswell, H. (1990). Birth and Population Increase from Matrix Population Models. In *Applied Mathematical Demography*, volume 46, pages 148–182. Springer-Verlag, New York.

Lefkovitch, L. P. (1965). The Study of Population Growth in Organisms Grouped by Stages. *Biometrics*, 21(1):1–18.

Perron, O. (1907). Zur Theorie der Matrices. *Mathematische Annalen 1*, 64(1):248–263.

Varga, R. S. (1962). *Matrix Iterative Analysis.* Springer.
