# Supplementary material for: Forest management affects seasonal source-sink dynamics in a territorial, group-living bird
Source: Oecologia. 2021 Jun 1;196(2):399–412. doi: 10.1007/s00442-021-04935-6 (PMC8241677; doi:10.1007/s00442-021-04935-6)
Supplement: Supplementary file 2 — Supplementary file1 (DOCX 660 kb) [file 442_2021_4935_MOESM2_ESM.docx]

**Supplementary material (Appendix 1 - 4)**

**Appendix S1.**

**Table S1.** The number of monitored territories, average group size per territory and the total number of individuals observed per year and forest type (natural or managed).

| **Year** |  | | **Number of**  **territories** | **Average**  **group size** | **Total number**  **of individuals** |
| --- | --- | --- | --- | --- | --- |
|  | | **Managed forest** | | | |
| 2000 |  | | 32 | 3.57 | 703 |
| 2001 |  | | 30 | 3.39 | 621 |
| 2002 |  | | 28 | 2.99 | 439 |
| 2003 |  | | 25 | 3.35 | 465 |
| 2004 |  | | 24 | 3.46 | 512 |
| 2005 |  | | 27 | 2.97 | 404 |
| 2006 |  | | 25 | 3.11 | 389 |
| 2007 |  | | 24 | 3.00 | 375 |
| 2008 |  | | 26 | 3.50 | 529 |
| 2009 |  | | 26 | 3.59 | 596 |
| 2010 |  | | 28 | 3.49 | 566 |
| 2011 |  | | 34 | 3.95 | 924 |
| 2012 |  | | 35 | 3.80 | 874 |
| 2013 |  | | 30 | 2.99 | 460 |
| 2014 |  | | 32 | 3.39 | 604 |
|  | | **Natural forest** | | | |
| 2000 |  | | 8 | 3.62 | 152 |
| 2001 |  | | 16 | 2.61 | 162 |
| 2002 |  | | 17 | 3.35 | 322 |
| 2003 |  | | 17 | 4.14 | 538 |
| 2004 |  | | 19 | 4.33 | 663 |
| 2005 |  | | 19 | 2.96 | 296 |
| 2006 |  | | 19 | 3.08 | 308 |
| 2007 |  | | 22 | 3.03 | 355 |
| 2008 |  | | 20 | 3.81 | 526 |
| 2009 |  | | 20 | 3.82 | 520 |
| 2010 |  | | 24 | 3.07 | 353 |
| 2011 |  | | 28 | 3.64 | 667 |
| 2012 |  | | 27 | 3.93 | 724 |
| 2013 |  | | 27 | 2.64 | 338 |
| 2014 |  | | 27 | 4.07 | 769 |

**Appendix S2.** Parameter estimates for vital rates in natural and managed forest.


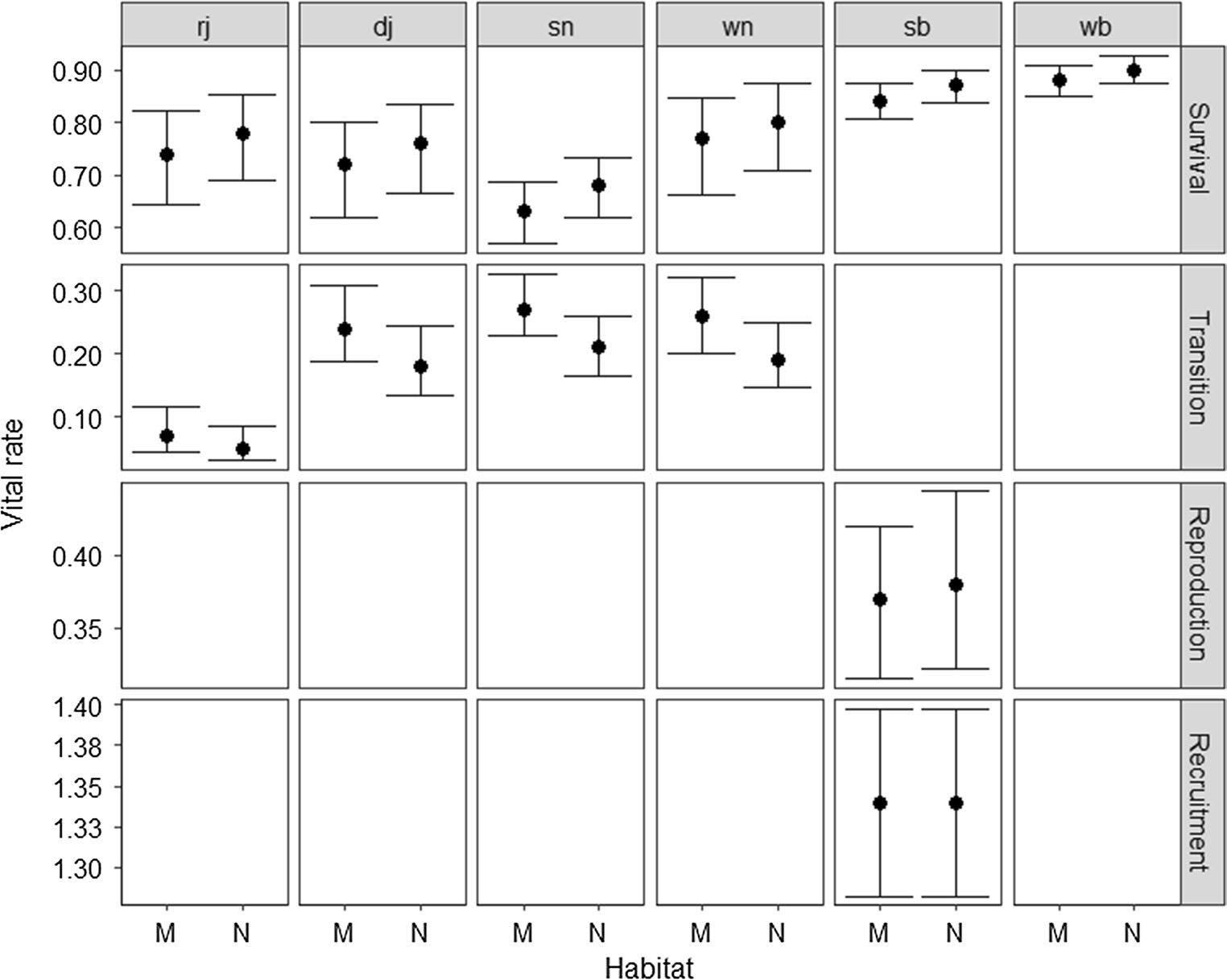


**Figure S1.** Survival (row 1), transition (row 2), reproductive (row 3) and recruitment rates (row 4) in managed (M) and natural (N) forest, for life history stages; retained juvenile (*rj*), dispersed juvenile (*dj*), summer non-breeder (*sn*), winter non-breeder (*wn*), summer breeder (*sb*) and winter breeder (*wb*) (from, Layton-Matthews, Ozgul, & Griesser, 2018).

**References**

Layton-Matthews, K., Ozgul, A. & Griesser, M. 2018. The interacting effects of forestry and climate change on the demography of a group-living bird population. *Oecologia***,** 1-12.

**Appendix S3.** Model selection for dispersal rate and dispersal distance.

**Table S1** Model selection table for dispersal rate

| **Model** | **npar** | **ΔAIC** |
| --- | --- | --- |
| habitat + stage | 7 | 0.0 |
| habitat + stage + habitat:stage | 12 | 2.4 |
| stage | 6 | 4.4 |
| habitat | 2 | 1100.2 |
| intercept | 1 | 1101.8 |

**Table S2** Model selection table with equations for the five dispersal kernels with associated ΔAIC values

| **Dispersal kernel** | **Equation** | **ΔAIC** |
| --- | --- | --- |
| Lognormal | $\text{f(x) = }\frac{\text{1}}{\text{x}\sqrt{\text{❑}}}$ | 0 |
| Weibull | $\text{f(x) = \{}{\frac{\frac{\text{k}}{\text{λ}}}{\text{0}}\text{(}\frac{\text{x}}{\text{λ}}\text{)}}^{\text{k-1}}\text{e}^{\text{-}{\text{(}\frac{\text{x}}{\text{λ}}\text{)}}^{\text{k}}}\frac{\text{x≥0}}{\text{x<0}}\text{\}}$ | 246 |
| Exponential | $\text{f(x) = λ}\text{e}^{\text{-λx}}$ | 388.2 |
| Gamma | $\text{f(x) = }\frac{\text{1}}{\text{Γ(k)}\text{θ}^{\text{k}}}\text{x}^{\text{k-1}}\text{e}^{\frac{\text{-x}}{\text{θ}}}$ | 816.6 |
| Gaussian | $\text{f(x) = }\frac{\text{1}}{\text{σ}\sqrt{\text{❑}}}$ | 1372.1 |

**Figure S1.** Estimates and associated 95% confidence intervals of stage, for summer juvenile (*sj*), summer and winter non-breeder (*sn*, *wn*) and summer and winter breeder (*sb*, *wb*), and habitat-specific (managed and natural) dispersal rates based on the best approximating model of dispersal rates (*Pd*).


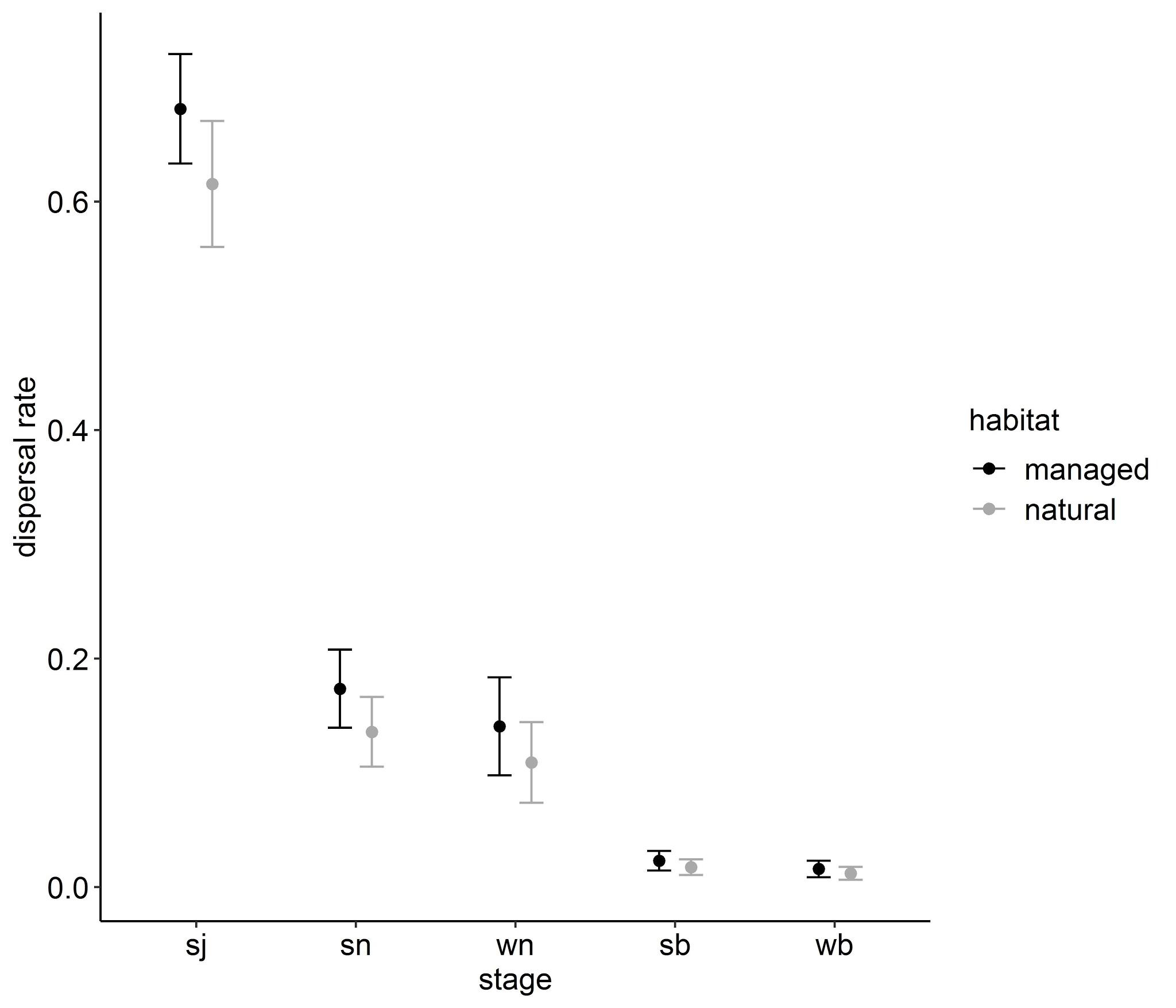

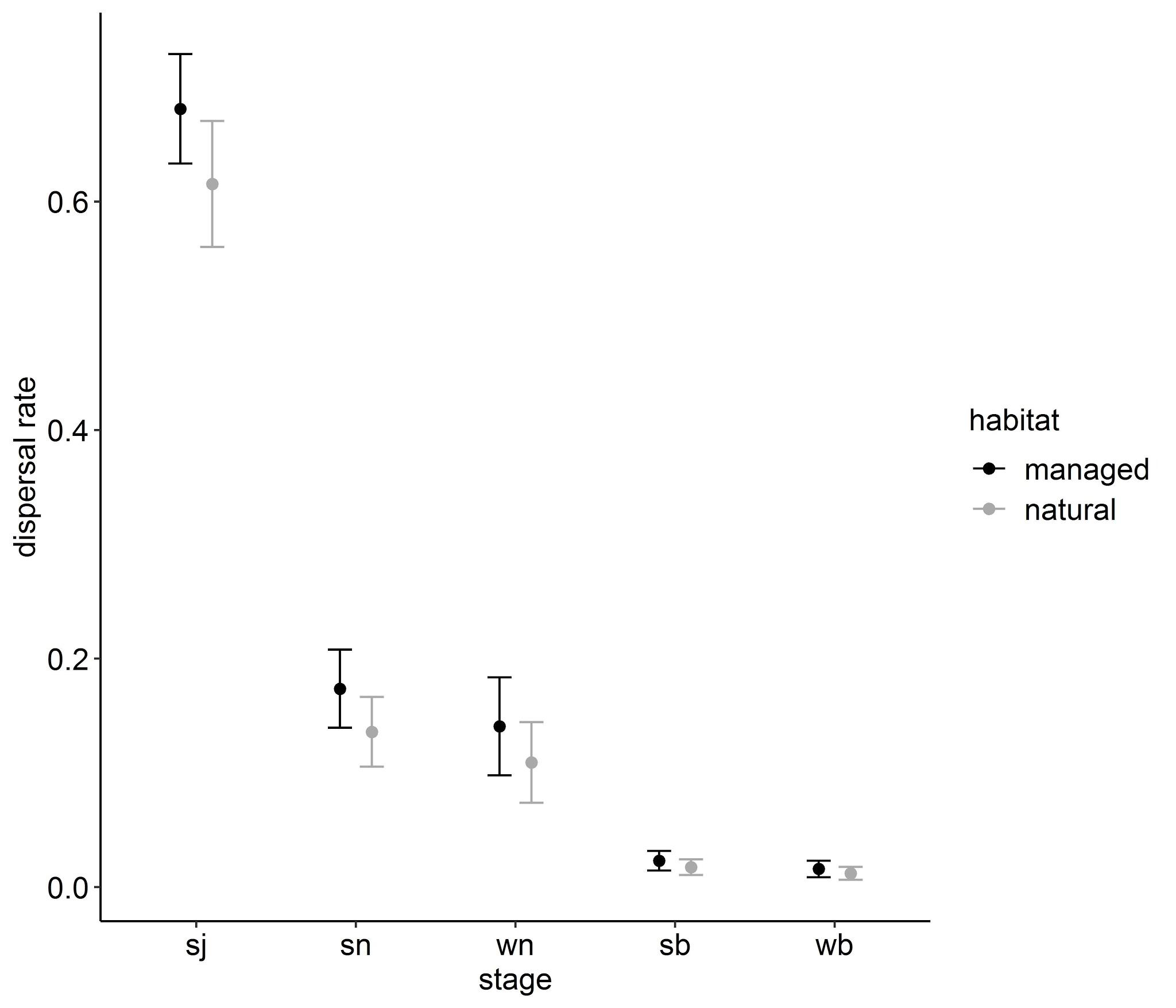


**Appendix S4**

Equations for the sensitivities of A*_MP_* to each of the seasonal (winter and summer) demographic and dispersal matrices.


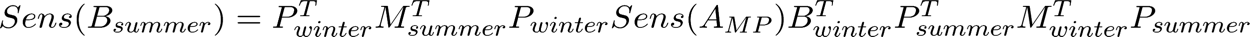


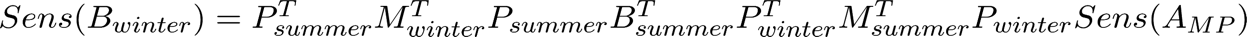


^
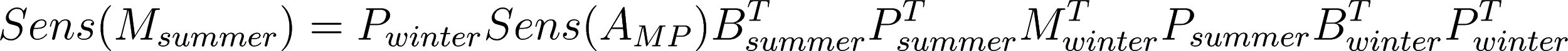
^


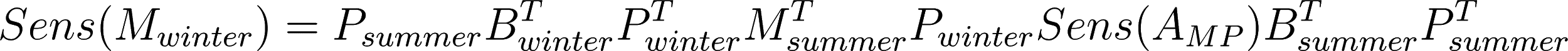


Proportional sensitivities or elasticities to the demography and dispersal matrices were then calculated as:


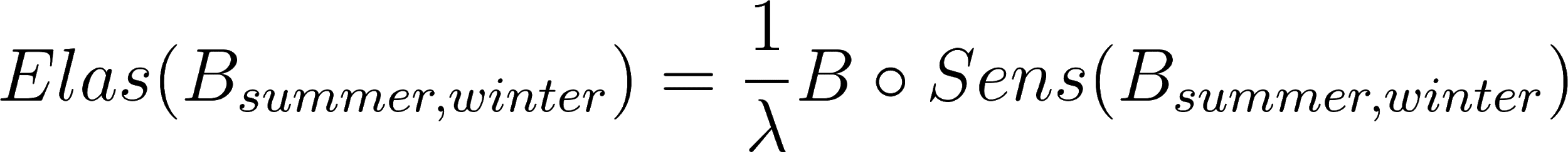


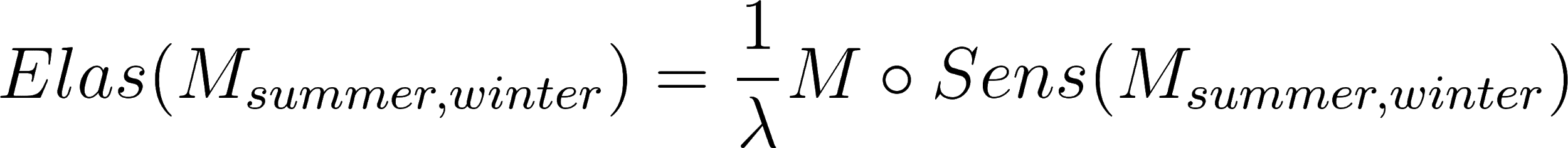


where ⚬ denotes the Hadamard product (Lesnoff, Ezanno, & Caswell, 2003).

**References**

Lesnoff, M., Ezanno, P. & Caswell, H. 2003. Sensitivity analysis in periodic matrix models: a postscript to Caswell and Trevisan. *Mathematical and Computer Modelling,* 37**,** 945-948.
